# Acquired reactive perforating dermatosis successfully treated with allopurinol^⋆^

**DOI:** 10.1016/j.abd.2022.06.009

**Published:** 2023-09-16

**Authors:** Ana Gusmão Palmeiro, Maria João Gonçalves, Cristina Amaro, Isabel Viana

**Affiliations:** a Dermatology Department, Hospital de Egas Moniz, Centro de Lisboa Ocidental, Lisbon, Portugal; b Rheumatology Department, Hospital de Egas Moniz, Centro de Lisboa Ocidental, Lisbon, Portugal

*Dear Editor,*

Acquired Reactive Perforating Dermatosis (ARPD) is a rare group of skin disorders, characterized by transepidermal elimination of various materials, such as collagen, elastin, and keratin. When the elimination occurs through a follicular unit, it is called perforating folliculitis.^1^ The most common systemic diseases associated with ARPD are diabetes mellitus and renal failure, but associations with infectious, inflammatory and autoimmune diseases have been reported, with possible triggers including immunological vasculopathy in the setting of the underlying autoimmune disorder and dermal microangiopathy due to scratching.

We present the case of a 54-year-old woman that was referred to the Dermatology outpatient clinic with a pruritic dermatosis of unknown evolution time. The dermatosis was diffusely distributed bilaterally on her thighs and elbows and was composed of erythematous-violaceus umbilicated papules with central adherent hyperkeratotic plugs (Fig. 1). Koebner phenomenon was present in some of the lesions. The patient had Rheumatoid Arthritis (RA), positive for both rheumatoid factor and anti-citrullinated protein antibodies, with unsatisfactory control under 12.5 mg of prednisolone daily. She had never been treated with biological drugs and had recently suspended sulfasalazine due to hepatotoxicity. Blood work revealed a mildly elevated erythrocyte sedimentation rate of 42 mm/h (normal <29 mm/h), but glycemia and kidney function were normal. Considering the cutaneous manifestations associated with rheumatoid arthritis, such as rheumatoid neutrophilic dermatosis and interstitial granulomatosis dermatitis, a punch biopsy was carried out. Histopathology revealed a dilated hair follicle with infundibular disruption, containing keratinous debris and neutrophils. Verhoeff stain evidenced that the eliminated materials were both elastic fibers and collagen (Fig. 2). Consequently, a diagnosis of perforating folliculitis was made.

**Figure 1:**
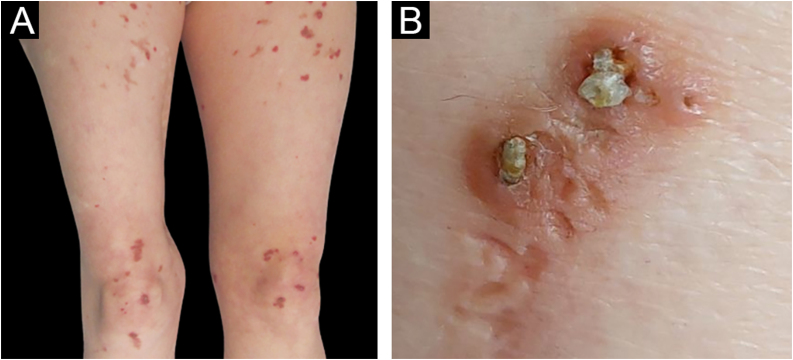
(A) Erythematous papules on the anterior surface of thighs and knees; (B) detail of central adherent hyperkeratotic plugs

**Figure 2:**
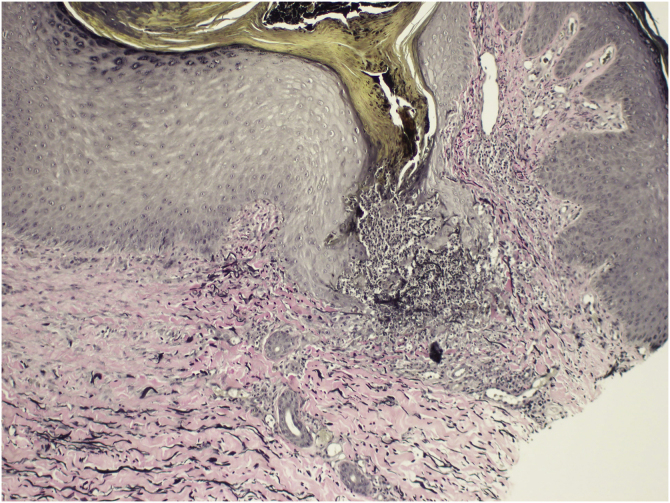
Histopathology of punch biopsy showing transepidermal elimination of elastic fibers (shown in black) and collagen (eosinophilic structures) (Verhoeff stain, ×100)

After a suboptimal response to topical clobetasol propionate and antihistamines and given the impossibility of safely undergoing phototherapy (the patient had a hearing and cognitive impairment), initiation of allopurinol 100 mg/day resulted in a considerable symptomatic relief and almost complete resolution of the dermatosis, with no adverse effects. The patient maintains regular follow at Dermatology and Rheumatology outpatient clinics and has since started abatacept, a disease-modifying anti-rheumatic agent.

As of today, there are no specific evidence-based guidelines regarding the treatment of perforating dermatosis.^2^ When possible, the priority should be the control of the underlying disease. Other treatment options include topical or systemic retinoids, antibiotics, topical, intralesional, or systemic glucocorticoids, antihistamines, surgery, narrow-band ultraviolet B phototherapy, PUVA and allopurinol. Allopurinol may be a valid and helpful treatment for ARPD, with successful results reported in 14 case reports and case series, with no reported adverse reactions.^2^, ^3^, ^4^ Its exact mechanism of action is unknown, but it may act via its antioxidant effect due to the inhibition of xanthine oxidase. A recent systematic review suggests it as a first-line systemic treatment, along with antihistamines.^2^

In conclusion, due to the rarity of these diseases and the lack of guidelines, the management of ARPD can be challenging. We present a rare association of perforating folliculitis and rheumatoid arthritis, successfully treated with allopurinol 100 mg once per day. Our experience supports allopurinol as a safe and effective treatment for ARPD.

## Financial support

None declared.

## Authors’ contributions

Ana Gusmão Palmeiro: Critical literature review; intellectual participation in propaedeutic and/or therapeutic; management of studied cases; data collection, analysis and interpretation; preparation and writing of the manuscript; study conception and planning.

Maria João Gonçalves: Manuscript critical review; intellectual participation in propaedeutic and/or therapeutic; management of studied cases.

Cristina Amaro: Manuscript critical review; approval of the final version of the manuscript; intellectual participation in propaedeutic and/or therapeutic; management of studied cases; study conception and planning.

Isabel Viana: Approval of the final version of the manuscript; intellectual participation in propaedeutic and/or therapeutic; management of studied cases.

## Conflicts of interest

None declared.
